# Various Kinds of Functional Digestive Tract Reconstruction Methods After Proximal Gastrectomy

**DOI:** 10.3389/fonc.2021.685717

**Published:** 2021-08-03

**Authors:** Shuaibing Lu, Fei Ma, Zhandong Zhang, Liangqun Peng, Wei Yang, Junhui Chai, Chen Liu, Fusheng Ge, Sheqing Ji, Suxia Luo, Xiaobing Chen, Yawei Hua

**Affiliations:** ^1^Department of General Surgery, The Affiliated Tumor Hospital of Zhengzhou University, Zhengzhou, China; ^2^Department of Medical Oncology, The Affiliated Tumor Hospital of Zhengzhou University, Zhengzhou, China

**Keywords:** digestive tract reconstruction, complications, reflux esophagitis, proximal gastric cancer, proximal gastrectomy, adenocarcinoma of esophagogastric junction

## Abstract

The incidence of proximal gastric cancer has shown a rising trend in recent years. Surgery is still the main way to cure proximal gastric cancer. Total gastrectomy with D2 lymph node dissection was considered to be the standard procedure for proximal gastric cancer in the past several decades. However, in recent years, many studies have confirmed that proximal gastrectomy can preserve part of the stomach function and can result in a better quality of life of the patient than total gastrectomy. Therefore, proximal gastrectomy is increasingly used in patients with proximal gastric cancer. Unfortunately, there are some concerns after proximal gastrectomy with traditional esophagogastrostomy. For example, the incidence of reflux esophagitis in patients who underwent proximal gastrectomy with traditional esophagogastrostomy is significantly higher than those patients who underwent total gastrectomy. To solve those problems, various functional digestive tract reconstruction methods after proximal gastrectomy have been proposed gradually. In order to provide some help for clinical treatment, in this article, we reviewed relevant literature and new clinical developments to compare various kinds of functional digestive tract reconstruction methods after proximal gastrectomy mainly from perioperative outcomes, postoperative quality of life and survival outcomes aspects. After comparison and discussion, we drew the conclusion that various functional reconstruction methods have their own advantages and disadvantages; large scale high-level clinical studies are needed to choose an ideal reconstruction method in the future. Besides, in clinical practice, surgeons should consider the condition of the patient for individualized selection of the most appropriate reconstruction method.

## Introduction

According to the global cancer statistics for 2020 (1) released by the International Agency for Research on Cancer of the World Health Organization, the incidence and mortality of gastric cancer ranked 5th and 4th, respectively among all malignant tumors on a global scale. In the past half century, although the incidence of distal gastric cancer has decreased in most regions, many surveys have indicated that the incidence of proximal gastric cancer has increased gradually (2, 3). To date, surgery is still the main way to cure early stage proximal gastric cancer. For proximal gastric cancer which includes the upper third stomach tumor and Siewert III adenocarcinoma of the esophagogastric junction, total gastrectomy (TG) with D2 lymph node dissection was considered a standard surgery in the past several decades (4). However, in recent years, many studies have concluded that proximal gastrectomy (PG) can preserve part of the stomach function, therefore, resulting in a better quality of life (QOL) of the patient than TG (5–7). Unfortunately, there are also some real concerns after PG with traditional esophagogastrostomy (EG); PG destroys the normal anatomical structure of esophagogastric junction and anti-reflux barrier (including structures such as the lower esophageal sphincter and diaphragmatic crura). At the same time, the preservation of pylorus delays gastric emptying. As a result, the incidence of reflux esophagitis (RE) and anastomotic related complications after PG is significantly higher than that after TG (7).

To solve those problems, various kinds of functional digestive tract reconstruction methods after PG have been proposed gradually. However, all of those methods have advantages and disadvantages. There is still a great controversy as to which reconstruction method can achieve optimal outcomes after PG. In order to provide some help for clinical treatment, in this article, we combined relevant literature and new clinical developments to review and compared digestive tract reconstruction methods after PG mainly from intraoperative status and postoperative complication aspects.

## Proximal Gastrectomy

According to the 5th edition of the Japanese Gastric Cancer Diagnosis and Treatment Guidelines (8) issued by the Japanese Gastric Cancer Association in 2018, PG is defined as the resection of part of the stomach including the cardia (esophagus–gastric junction), while preserving pylorus under the premise of radical tumor resection. For patients with early stage (cT1N0) proximal gastric cancer, PG with more than 1/2 of the distal stomach preserved and D1/D1+ lymph node dissection is feasible under the condition of ensuring a safe margin.

Currently, the main problem after PG is RE. The sphincter structure at the esophagogastric junction includes the lower esophageal sphincter composed of smooth muscle and the crura of diaphragm composed of skeletal muscle, which overlaps and jointly maintains the pressure of lower esophagus (9). PG destroys the normal anatomical structure of esophagogastric junction and anti-reflux barrier which include structures such as the lower esophageal sphincter and diaphragmatic crura. At the same time, the preservation of pylorus delays gastric emptying. As a result, the incidence of reflux esophagitis and anastomotic related complications is significantly higher in proximal gastrectomy patients than in total gastrectomy patients. Various kinds of functional digestive tract reconstruction methods after PG are trying to solve those problems.

## Surgical Procedure of Various Reconstruction Methods After Proximal Gastrectomy

According to the 5th edition of the Japanese Gastric Cancer Diagnosis and Treatment Guidelines issued by the Japanese Gastric Cancer Association in 2018 (8), there are three recommended methods to reconstruct digestive tract after PG: esophagogastrostomy (EG), jejunal interposition (JI), and double tract reconstruction (DTR). On the basis of these three reconstruction methods, a variety of digestive tract reconstruction methods have been proposed in recent years.

The methods derived from traditional EG include: tube-like stomach esophagogastrostomy (tube-like stomach EG), double-flap technique (DFT), and side overlap with fundoplication by Yamashita (SOFY), *etc.* Jejunal pouch interposition (JPI) derived from JI. The methods derived from DTR include modified double tract reconstruction (modified DTR). Furthermore, there are some techniques to prevent reflux, such as preservation of lower esophageal sphincter, vagus nerve preservation, and pyloroplasty.

### Traditional Digestive Tract Reconstruction Method

#### Traditional Esophagogastrostomy

**Figure 1:**
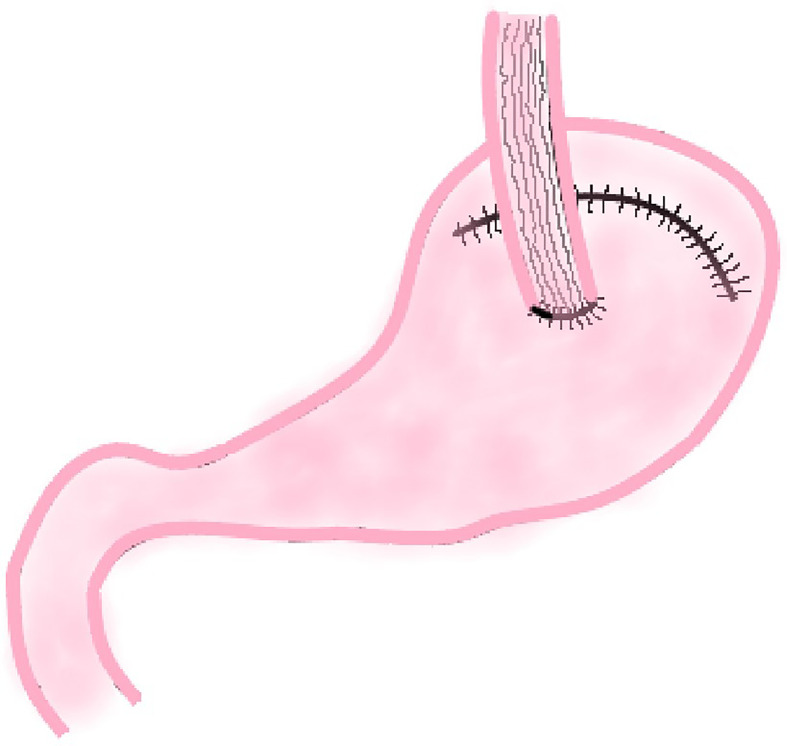
Traditional Esophagogastrostomy.

The traditional EG involves an end-to-side anastomosis of the esophagus and the anterior or posterior wall of the remnant stomach or end-to-end anastomosis of esophagus and remnant stomach (**Figure 1**). Related research suggests that these three methods have similar 2-year survival rates (P = 0.713) and surgical results, but the end-to-side anastomosis between esophagus and the anterior wall of the remnant stomach results in a better postoperative quality of life than the other two methods, which is reflected in faster weight recovery, less discomfort after meals, and less heart burn or belching at 6 and 24 months postoperatively (10).

The reason for this result may be that the esophagogastric anterior wall anastomosis is above the remnant stomach in supine position and the lower stomach contents and stomach acid are not easy to reflux into the esophagus. The anastomosis between esophagus and the posterior wall of the remnant stomach is just on the contrary. As a result, end-to-side anastomosis of esophagus and anterior wall of the remnant stomach is more common in clinical practice.

### Functional Digestive Tract Reconstruction Methods

At present, the common functional digestive tract reconstruction methods after PG can be divided into two categories. One is direct anastomosis between esophagus and remnant stomach, and the other is interposition of jejunum between esophagus and remnant stomach. The former includes tube-like stomach EG, DFT, and SOFY. The later includes JI, JPI, DTR, and modified DTR.

#### Direct Anastomosis Between Esophagus and Remnant Stomach

##### Tube-Like Stomach Esophagogastrostomy

**Figure 2:**
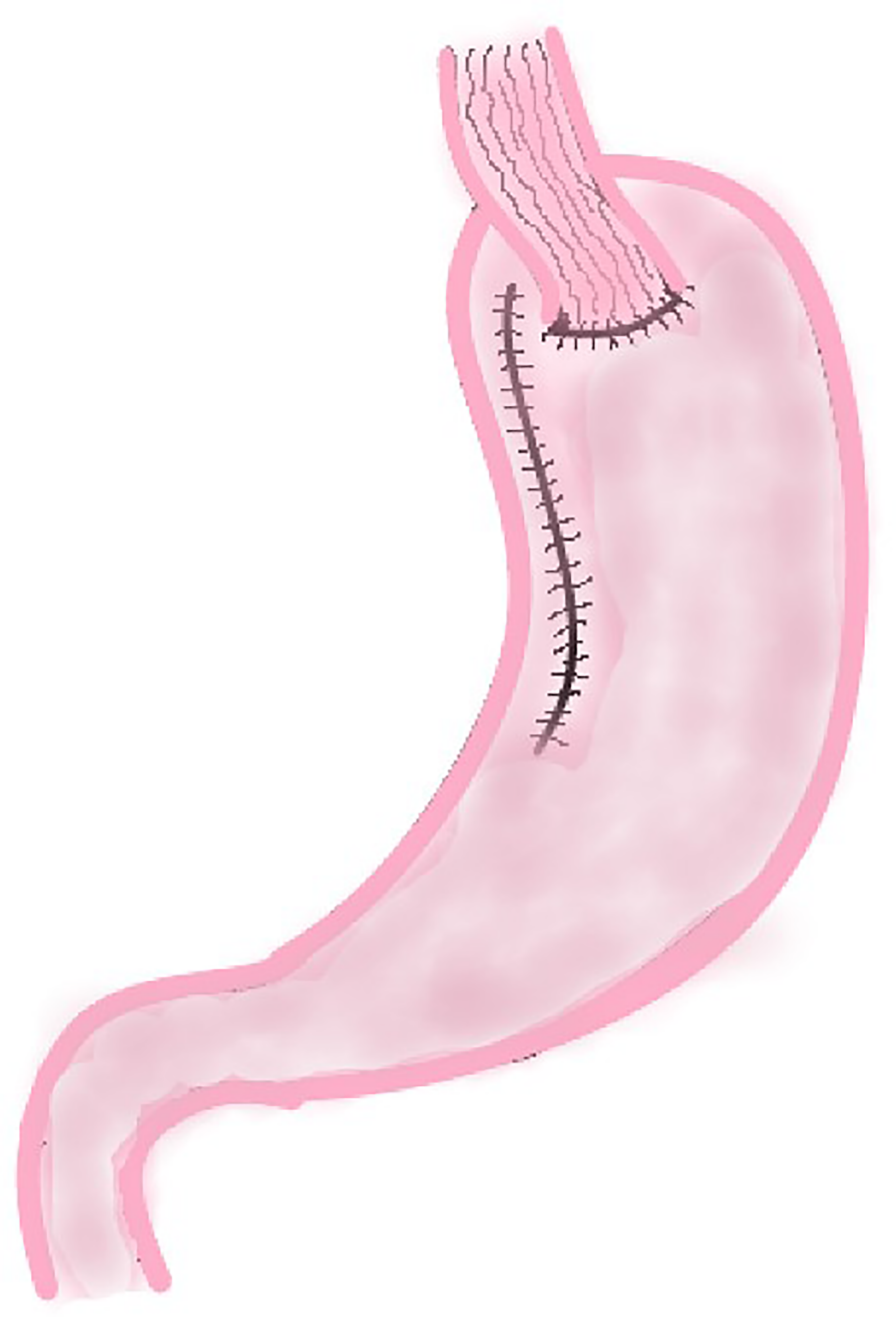
Tube-like Stomach Esophagogastrostomy.

This method was first reported by Shiraishi et al. in 1998 and was used to prevent reflux esophagitis after proximal gastrectomy (11). In this procedure, surgeons use a linear cutting suture device to make a curve parallel to the greater curvature of the stomach (approximately 4.0 cm from the greater curvature of the stomach) along the lesser curvature of the stomach to remove the cardia, tumor, and part of the lesser curvature of the stomach. After this procedure, a length of about 20 cm gastric tube is created, and then end-to-side esophagogastric anastomosis is performed (**Figure 2**).

##### Double-Flap Technique

**Figure 3:**
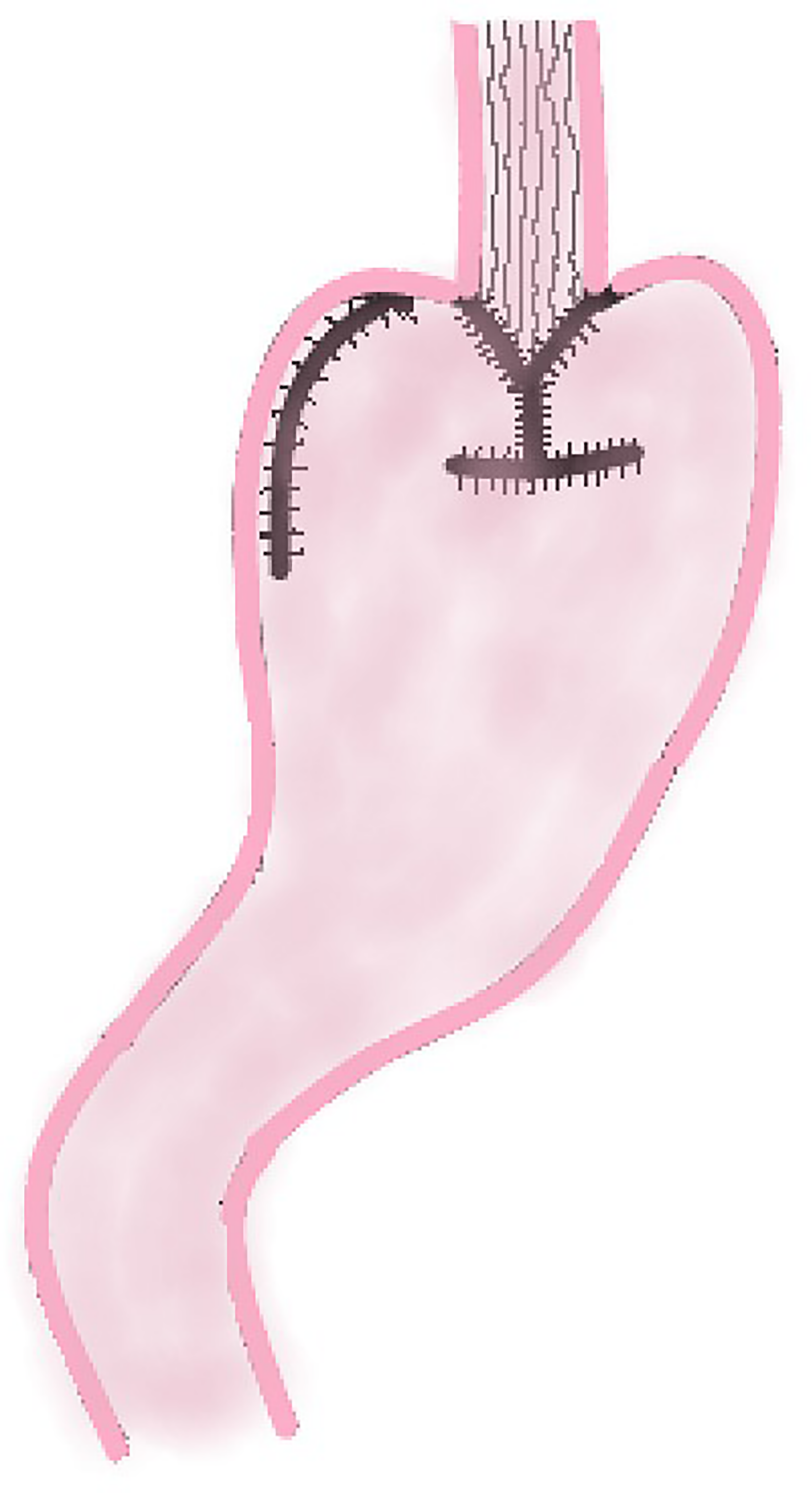
Double Flap Technique.

DFT is to incise the muscle layer of the anterior wall of the remnant stomach at approximately 4.0 cm below the margin of the remnant stomach after PG, and make double seromuscular flaps (approximately 2.5 cm × 3.5 cm). Then the mucosa and submucosa are incised at the lower edge of the window formed by the seromuscular flap, and the esophageal stump is anastomosed with the mucosa and submucosa. Finally the two seromuscular flaps are covered on the lower esophagus and the upper layer of the anastomosis (**Figure 3**). This method increases the pressure of lower esophagus because the covered muscle flap has a tension on lower esophagus, which is conducive to reduce the occurrence of RE.

##### Side Overlap With Fundoplication by Yamashita

SOFY was first reported by Japanese scholar Yamashita in 2016 (12). This procedure fixes the remnant stomach at the left and right diaphragmatic crura to reconstruct the artificial fundus of the stomach; then, the left side of the esophageal stump is anastomosed with the anterior wall of remnant stomach side-to-side, the linear stapler is inserted into esophagus, and stomach is rotated counterclockwise and fixed to ensure stomach wall is sutured on the side wall of the esophagus. And the opposite side wall of esophagus is fixed to stomach so that the esophagus is close to the stomach wall. When the pressure of the artificial fundus of stomach increases, the anastomotic stoma is closed, thus acting as an anti-reflux function. It is considered that this reflux prevention mechanism is a consequence of the combination of valvuloplasty and fundoplication.

#### Interposition of Jejunum Between Esophagus and Remnant Stomach

##### Jejunal Interposition

**Figure 4:**
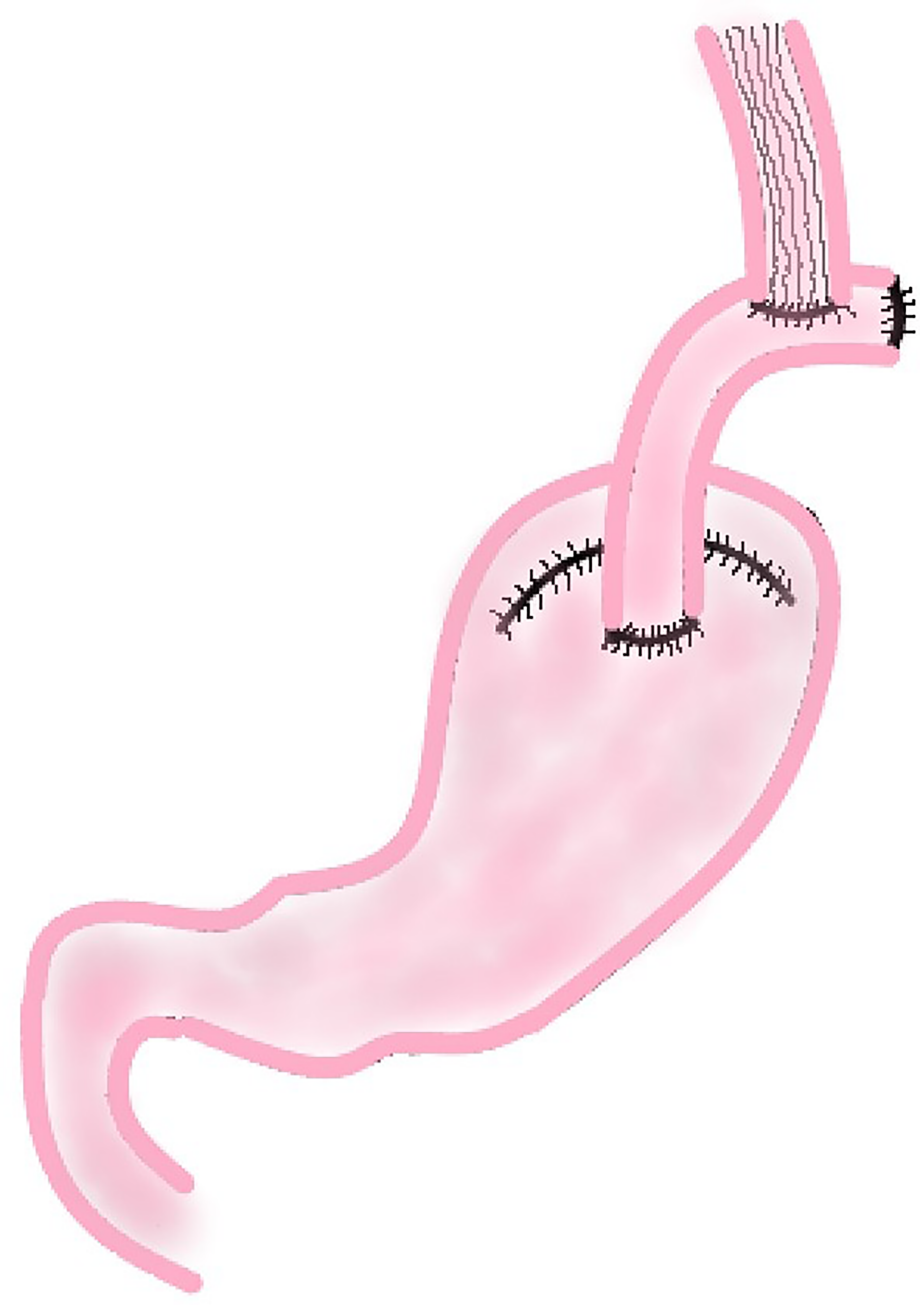
Jejunal Interposition.

In this procedure, a section of jejunum with vascular pedicle is cut and anastomosed with esophageal stump and the anterior wall of the remnant stomach respectively. Then the proximal jejunum and the distal jejunum are anastomosed to reconstruct the digestive tract (**Figure 4**). This method not only increases the capacity of the remnant stomach, but using the jejunum itself, also tolerates stomach acid and natural peristalsis to build an anti-reflux barrier. A section of jejunum is placed between esophagus and stomach, which reduces the tension of the anastomosis and be highly safe.

##### Jejunal Pouch Interposition

**Figure 5:**
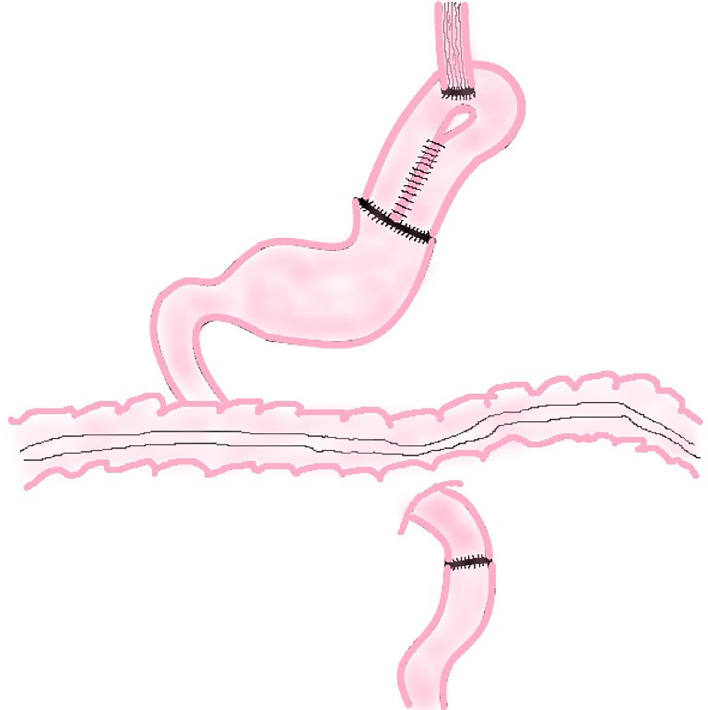
Jejunal pouch Interposition.

The basic principle of JPI is similar to JI. The only difference is that JPI needs to cut about 25 cm long jejunum and fold it side by side to make a 10 cm long inverted U-shaped or P-shaped jejunum pouch. Then the jejunum pouch is anastomosed side-to-end with the esophagus and end-to-side with the remnant stomach (**Figure 5**). It should be noted that the U-shaped loop of the jejunal pouch (about 3 cm) does not anastomose and remains intact to form a septum to maintain the blood flow at the anastomosis between the esophagus and the pouch and prevent reflux.

##### Double Tract Reconstruction

**Figure 6:**
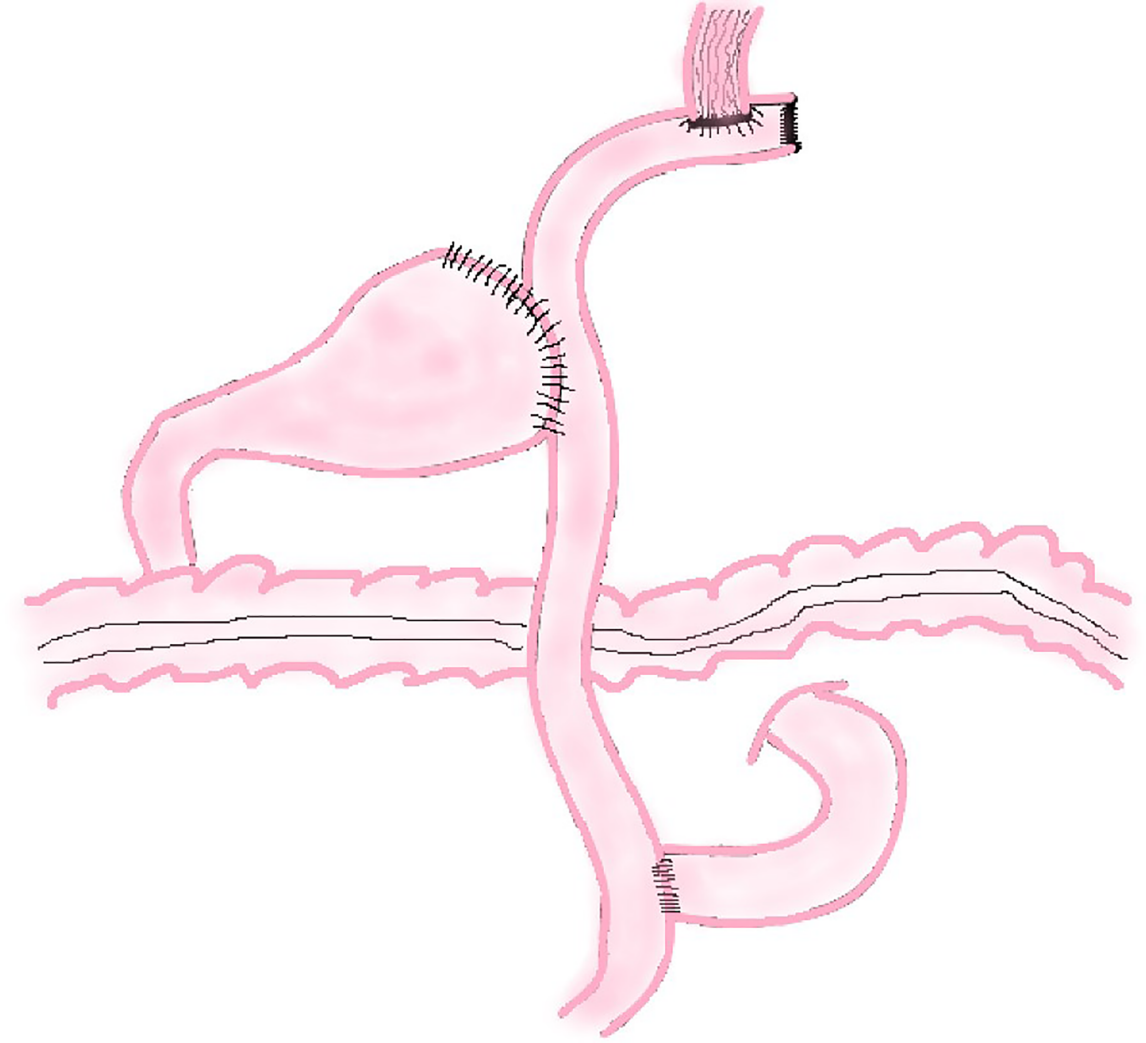
Double Tract Reconstruction.

In 1988, Aikou et al. first reported the application of DTR as a digestive tract reconstruction method for proximal gastrectomy (13). After cutting off the jejunum and mesangial vessels at 20–25 cm away from the ligament of Treitz, the esophagus is anastomosed with the distal jejunum, and the stump of the jejunum is closed with a linear stapler; The jejunum at 10–15 cm from the esophageal–jejunal anastomosis is anastomosed with the anterior wall of the remnant stomach side-to-side, and the stomach stump is closed; the distal jejunum at a distance of 45–50 cm from the esophageal–jejunal anastomosis is anastomosed with the proximal jejunum (**Figure 6**).

##### Modified Double Tract Reconstruction

**Figure 7:**
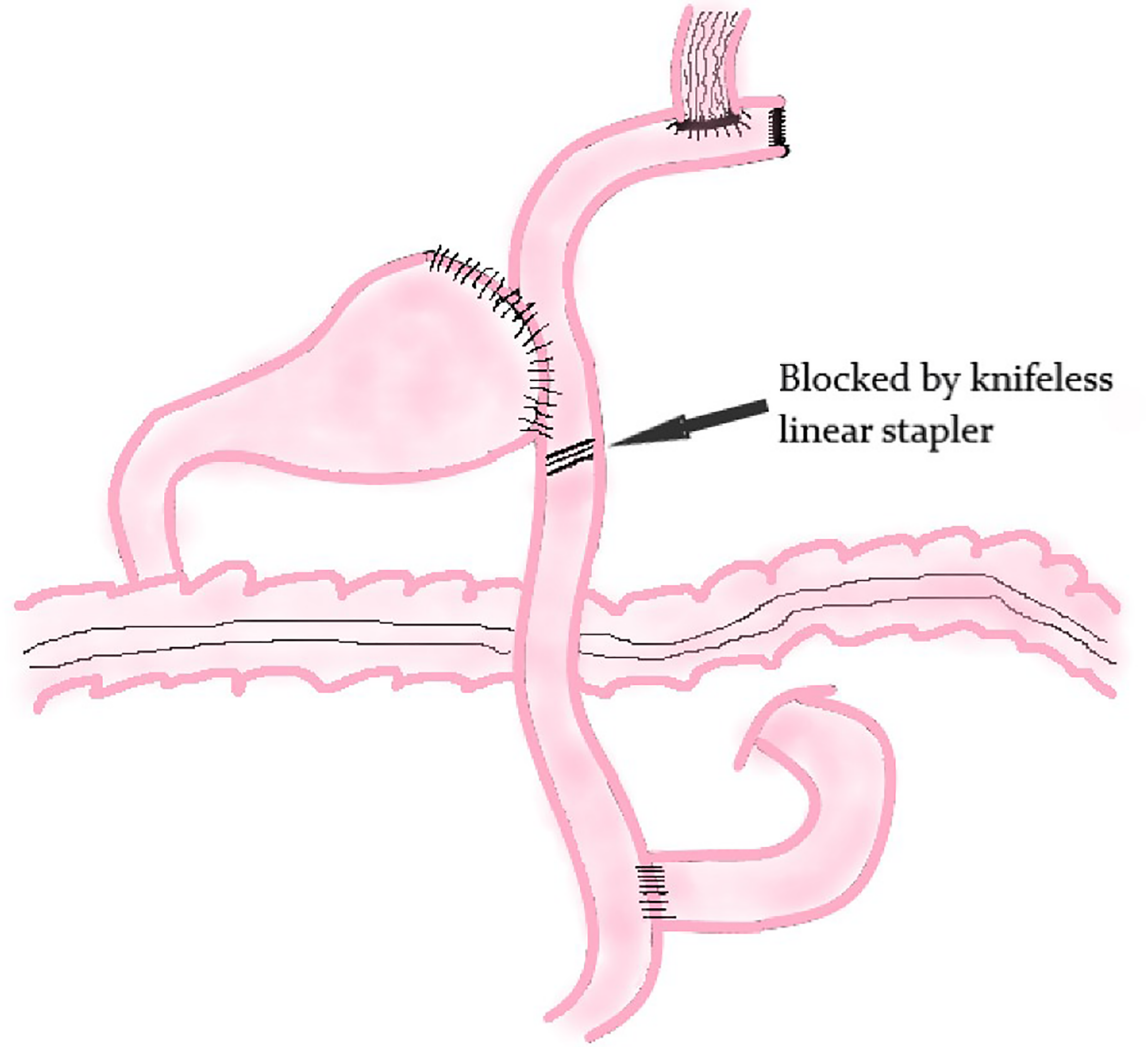
Modified Double Tract Reconstruction.

The principle of modified DTR is based on DTR. The difference between these two methods is that a knifeless linear stapler is used to block the distal jejunum pathway below the jejunum stump in modified DTR (**Figure 7**); it means that this digestive tract reconstruction method does not cut off the jejunum segment.

## Clinical Outcomes of Various Reconstruction Methods After Proximal Gastrectomy

In recent years, many studies have concluded that proximal gastrectomy (PG) can preserve part of stomach function, therefore, results in a better quality of life (QOL) than TG. The results of several studies on the comparison of perioperative outcomes and postoperative nutritional outcomes of PG and TG are listed in **Table 1**. The main differences between these two methods are intraoperative parameters and postoperative nutritional outcomes. PG has shorter operation time (7, 14, 15) and less intraoperative bleeding (14, 15, 17) than TG. Compared with TG, PG has better postoperative nutritional outcomes such as weight maintenance, serum hemoglobin level, and albumin level due to its preservation of distal stomach (5, 7, 14–17). At present, it is generally accepted that PG results in a better postoperative quality of life (QOL) than TG.

**Table 1:** Perioperative outcomes and postoperative nutritional outcomes of PG v.s. TG.

Author	Surgical methods	Sex (m/f)	Intraoperative status		Complications				Nutritional outcomes		
			Operation time(min)	blood loss(ml)	Reflux esophagitis n(%)	Anastomosis stenosis n(%)	Anastomosis leakage n(%)	Total complications n(%)	Body weight	hemoglobin level	Serum albumin level
Ichikawa (5)	TG	29/6	\	\	\	1 (2.9)	1 (2.9)	4 (11.4)	Is PG group better than TG group ?	Is PG group better than TG group ?	Is PG group better than TG group ?
	PG	34/15	\	\	\	3 (6.1)	1 (2.0)	6 (12.2)			
	P-value	0.16	\	\	\	\	\	0.89	YES ^Δ^	YES ^Δ^	\
Yamasaki (7)	TG	67/26	296 (133–661)	170 (15–1266)	5 (5.0)	5 (5.0)	8 (9.0)	\	Is PG group better than TG group ?	Is PG group better than TG group ?	Is PG group better than TG group ?
	PG	116/43	244 (110–494)	180 (10–2379)	23 (14.0)	9 (6.0)	7 (4.0)	\			
	P-value	0.89	< 0.001	0.26	0.02	0.92	0.17	\	YES ^Δ^	YES ^Δ^	NO
Masuzawa (14)	TG	89/33	225 ± 41	368 ± 316	\	2 (1.6)	6 (4.9)	16 (13.1)	Is PG group better than TG group ?	Is PG group better than TG group ?	Is PG group better than TG group ?
	PG-EG	36/13	185 ± 48	280 ± 247	\	2 (4.1)	0 (0.0)	4 (8.2)			
	P-value	ns	<0.0001	0.0337	\	ns	\	ns	YES	YES	YES ^Δ^
Ahn (15)	TG	56/25	242.2 ± 52.5	181.7 ± 138.0	3 (3.7)^a^	4 (4.9)	\	18 (22.2)	Is PG group better than TG group ?	Is PG group better than TG group ?	Is PG group better than TG group ?
	PG	36/24	216.3 ± 56.0	115.8 ± 81.9	16 (32.0)^a^	6 (12.0)	\	22 (44.0)			
	P-value	0.728	0.009	0.002	<0.001	0.139	\	0.005	NO	YES	NO
Son (16)	TG	76/30	\	\	24 (22.6)	3 (2.8)	2 (1.9)	14 (13.2)	Is PG group better than TG group ?	Is PG group better than TG group ?	Is PG group better than TG group ?
	PG-GTR	43/21	\	\	6 (9.4)	1 (1.6)	0 (0.0)	3 (4.7)			
	P-value	0.605	\	\	0.028	0.597	0.269	0.186	YES ^Δ^	YES ^Δ^	YES ^Δ^
Tsumura (17)	TG	11/6	306(256-371)	70 (40–117.5)	1 (5.0)	0	1 (5.0)	2 (12.0)	Is PG group better than TG group ?	Is PG group better than TG group ?	Is PG group better than TG group ?
	PG	16/3	280 (264–345)	210 (90–285)	0	1 (5.0)	0	2 (11.0)			
	P-value	0.1773	0.6804	0.0106	\	\	\	0.9060	YES ^Δ^	\	\

^a^reflux symptom; ^Δ^significant difference(P<0.05); ns, no significant difference.

However, there is still a great controversy about which reconstruction method can achieve optimal outcomes after PG because each of the above reconstruction methods after PG has its merits and demerits. Data of various kinds of reconstruction methods after PG of part references included in this article are listed in **Table 2**. The inclusion criteria of studies listed in **Table 2** are as follows. 1) The published year ranged from 1996 to 2020. 2) Patients underwent PG for the upper third stomach cancer or adenocarcinoma of esophagogastric junction. 3) The number of patients was no less than 10 cases. 4) Study focused on intraoperative status and postoperative complications, such as reflux esophagitis and anastomotic stenosis.

**Table 2:** Outcomes of various kinds of reconstruction methods after proximal gastrectomy.

Type of anastomosis	Number of anastomoses	Author	Country	Year	Patients(m/f)	Operation time(min)	Blood loss(ml)	Reflux esophagitis n(%)	Los Angeles classification A/B/C/D(n)	Stenosis n(%)
traditional EG	1	Tokunaga (18)	Japan	2008	36(30/6)	195.8±8.2	294.2±55.4	11 (30.6)	3/0/6/2	\
		Nakamura (19)	Japan	2014	64(49/15)	198±25	179±158	15/55 (27.3)	3/8/4/0	12/55(21.8)
		Chen (20)	China	2012	41(32/9)	268.4±28.18	204.15±147.97	9 (22.0)	6/2/1/0	9(22.0)
tube-like stomach EG	1	Adachi (21)	Japan	1999	14	164.9±38.3	151.1±99.6	1 (7.1)	\	1(7.1)
		Chen (20)	China	2012	35(27/8)	280.0±25.48	209.71±163.45	2 (5.7)	2/0/0/0	4 (11.4)
DFT	1	Tsumura (17)	Japan	2020	19(16/3)	280 (264–345)	210 (90–285)	0 (0.0)	0/0/0/0	1(5.0)
		Shoji (22)	Japan	2019	147(113/34)	420 (244–641)	70 (10–670)	6 (4.2)	0/3/3/0	12(8.3)
		Kuroda (23)	Japan	2018	464(407/139)	298(247.5-370.5)	240(100-392.5)	49 (10.6)	21/20/6/2	26(5.5)
SOFY	1	Yamashita (12)	Japan	2017	14(11/3)	330 (273-361)	17.0 (10-40)	1/10 (10.0)	0/1/0/0	0 (0.0)
JI	3	Tokunaga (18)	Japan	2008	40(31/9)	256.5±10.2	299.3±35.4	2 (5.0)	2/0/0/0	\
		Nakamura (19)	Japan	2014	25(21/4)	281±69	393±338	1/22 (4.5)	1/0/0/0	7/22(31.8)
		Adachi (21)	Japan	1999	16	326.9±70.6	508.4±318.1	0 (0.0)	0/0/0/0	1(7.1)
JPI	3	Nakamura (19)	Japan	2014	12(9/3)	311±68	402±385	2/12 (16.7)	1/1/0/0	1/12(8.3)
		Yoo (24)	Korea	2005	25(21/4)	230.6±26.3	288.±102.3	0 (0.0)	\	1(4.0)
DTR	3	Hong (25)	China	2016	21(16/5)	173.8±21.8	109.2±96.3	0 (0.0)	\	0 (0.0)
		Ahn (26)	Korea	2014	43(35/8)	180.7±38.7	120.4±74.3	0 (0.0)	\	2(4.7)
		Nomura (27)	Japan	2014	10(8/2)	\	\	1 (10.0)	\	1(10.0)
		Nomura (28)	Japan	2018	15(13/2)	352.5±67.3	90.5±105.5	1 (6.7)	\	2(13.3)
modified-DTR	3	Nomura (27)	Japan	2014	10(7/3)	\	\	1 (10.0)	\	2(20.0)
		Nomura (28)	Japan	2018	15(11/4)	322.5±24.2	46.8±69.8	1 (6.7)	\	2(13.3)

### Traditional Esophagogastrostomy

Because anastomosis of esophagus is direct and remnant stomach has only one anastomosis, the operation is simple with less intraoperative blood loss, and complications related to anastomosis such as leakage and stenosis are relatively rare (18, 19). Based on the above advantages, traditional EG has a strong clinical feasibility. However, due to its lack of anti-reflux structure, the incidence of postoperative RE is significantly higher than other reconstruction methods (18, 19). Shaibu et al. conducted a statistical analysis on 29 research data and showed that the incidence of RE in EG, JI, JPI, DFT, and DTR was 19.3, 13.8, 13.8, 8.9, and 8.6%, respectively (29). For this reason, traditional EG has been rarely used alone; it is often used in combination with some anti-reflux measures such as preservation of the vagus nerve (30) and establishment of artificial fornix (19), which can prevent RE after surgery.

To date, consensus on whether routine pyloroplasty is needed after PG is yet to be established. Some scholars suggest that routine addition of pyloroplasty to patients with EG after PG can prevent the occurrence of delayed gastric emptying and improve postoperative quality of life obviously (10, 31, 32). Opponents argue that adding pyloroplasty will increase the incidence of dumping syndrome (33) and bile reflux esophagitis (34). In Velanovich’s study, they found that without pyloroplasty, the incidence of symptoms related to gastric emptying disorder is not high (5.2%) (35), but it can effectively prevent the occurrence of dumping syndrome and bile reflux esophagitis theoretically.

In recent years, laparoscopic gastrectomy has been widely carried out as a minimally invasive surgical method. The promotion of laparoscopic technology in PG gives EG more room for innovation, development, and improvement.

### Functional Digestive Tract Reconstruction Methods

#### Direct Anastomosis Between Esophagus and Remnant Stomach

##### Tube-Like Stomach Esophagogastrostomy

Adachi et al. conducted a study on this reconstruction method after PG in 1999 (21). The results showed that the operation time was short, intraoperative blood loss was less, and the postoperative hospital stay was short. In this study, only one patient had mild RE and one patient had anastomotic stenosis in 14 patients who underwent tube-like stomach EG, which verified tube-like stomach reconstruction after PG is not only simple and safe, but also has a considerable effect on preventing RE.

Compared with other reconstruction methods, the anatomical structure of the tube-like stomach has many theoretical advantages as follows: there is only one anastomosis so the operation is simple, easy and safe; tubular stomach extends the reflux distance and thus reduces the reflux symptoms; part of the gastric antrum area is removed so gastric acid secretion is significantly reduced; the smooth food passage contributes to the low incidence of delayed gastric emptying and RE. Additionally, compared to reconstruction by interposition of jejunum, the proximal jejunum which is important for digestion and absorption of nutrients is preserved. Thus, it can improve the nutritional status of patients after PG. However, Li et al. postulate that the application of tubular gastroesophageal anastomosis in radical PG has some defects (36). Excessive cutting and sutures may cause gastric bleeding, poor healing, and gastric fistula; the arterial blood supply and venous return of tubular stomach may be damaged; the gastric cavity of tubular stomach becomes smaller and the path which becomes straight may lead to an increase in the incidence of various complications such as abnormal emptying and reflux esophagitis; the tubular stomach easily leads to a significant increase in the tension of the anastomotic stoma, postoperative anastomotic leakage and stenosis. But for all that, more studies showing the advantages of tube-like stomach EG after PG are being reported. In recent years, several studies about tube-like stomach reconstruction after PG confirmed this method is simple, feasible, and patients have better postoperative QOL (20, 37). Therefore, it still deserves more clinical applications and warrants an in-depth research in the future.

##### Double-Flap Technique

At present, DFT has been widely carried out in clinical practice. A lot of studies have shown that this method is more effective in preventing postoperative RE and has a lower risk of anastomotic leakage (17, 22, 23, 38, 39). In those studies, it is worth noting that the muscle flap tension tended to be too high during the anastomosis theoretically, but the incidence of anastomotic stenosis was not too high (4.7–8.3%). It might be due to the use of intraoperative endoscope to preserve the anastomotic lumen (39) and the softness of the posterior side of anastomotic sites which comprised only mucosa (38).

DFT has not only been widely used in digestive tract reconstruction after open and laparoscopical PG, but has also been applied after robotic PG. In 2017, Shibasaki et al. performed robotic PG with DFT on 12 patients with proximal gastric cancer (40). In this study, they found that there was no case of RE; there were three cases of anastomotic stenosis (25%) in the 2 months of follow-up after operation, which was strongly related to the excessive number of suture stitches in the anastomosis of esophagus and remnant stomach during operation (P < 0.001). This study suggests robotic PG with DFT is effective in preventing reflux esophagitis. According to a research conducted by Son’s team (41), laparoscopic gastrectomy requires about 40–60 operations to achieve technical proficiency, while robotic gastrectomy requires about 20 or less operations to complete the learning curve. In Son’s study, six operations can reach the plateau of learning curve. In general, robotic PG with DFT is a safe and effective surgical method for proximal gastric cancer patients and can achieve a short learning curve for clinical surgeons. Therefore, robotic PG with DFT is a promising surgical method, and more improvement is needed to prevent postoperative anastomotic stenosis in the future.

##### Side Overlap With Fundoplication by Yamashita

This reconstruction method has just been proposed in 2016, so there is very little study on SOFY. According to Yamashita’s research, this procedure is simple, can be done well under laparoscopy, and may be able to overcome postoperative RE and anastomotic complications such as stenosis (12). In this study, only one patient had RE of Los Angeles classification grade B; no patient had anastomotic stenosis or leakage in 14 patients who underwent SOFY. This result is significantly better than the non-SOFY group: cases of reflux esophagitis, anastomotic stenosis and leakage were five, three, and two respectively. On the basis of this study, this method may have wide clinical application prospect, and we look forward to a large sample multi-center randomized controlled trial to generate more convincing data with regard to SOFY in the future.

#### Interposition of Jejunum Between Esophagus and Remnant Stomach

##### Jejunal Interposition

According to the results of a study conducted by Tokunaga which compared the outcomes of patients after PG with JI and PG with EG, there was no significant difference in the 5-year survival rate between the EG group (94.2%) and the JI group (96.9%). But the incidence of RE was significantly lower in the JI group than in the EG group (p = 0.001), and there was no severe reflux esophagitis (Los Angeles classification grade C or D) in the JI group (18). It might due to the interposed jejunum which functions as a buffer for gastric acid and protects the esophagus against gastric acid reflux. This conclusion is similar to the study conducted by Nakamura et al. (19). However, this method also has some disadvantages. For example, this reconstruction is more complicated than direct anastomosis between esophagus and remnant stomach, so the operation results in more intraoperative blood loss and needs longer time (18, 19, 21). Even so, with the gradual maturity of laparoscopic techniques, the clinical application of JI after laparoscopic PG has increased significantly in recent years. A study has shown that the safety and curative effect of JI after laparoscopic PG are basically the same as those of open surgery, while the laparoscopic group has less intraoperative bleeding, faster postoperative recovery, better short-term quality of life after surgery than the open group (42). This suggests that laparoscopic PG with JI may be a way to overcome the disadvantages of JI in the future.

At present, the main controversy about JI is the length of the interposed jejunum. Most scholars advocate that the length of the interposed jejunum is 10 cm or 15 cm. It is generally presumed that the shorter the interposed jejunum is, the smoother will be the endoscopic diagnosis and treatment after surgery; the longer the interposed jejunum is, the better the effect it will have on preventing RE (43, 44). However, in the study of Tokunagas (18), they did a subgroup analysis on patients with the length of interposed jejunum >10 cm and the length of interposed jejunum ≤10 cm. Except for the shorter operation time (p = 0.02) and exploration of remnant stomach with postoperative endoscopy was more convenient in the ≤10 cm group of patients, no significant difference was found in the incidence of RE and other postoperative complications. Therefore, the author postulates that an interposed jejunum of 10 cm or shorter was sufficient to prevent RE and a shorter jejunum segment is beneficial to postoperative evaluation of remnant stomach. Because these studies involved only a small number of patients, a large sample prospective randomized controlled study is needed to guide clinical practice with regard to the optimal length of the interposed jejunum after PG.

Whether JI will be the mainstream reconstruction method after PG in the future is still inconclusive. Although the present study shows some disadvantages of JI about operation time and intraoperative blood loss, it should be a prior choice for digestive tract reconstruction for it could effectively prevent severe RE. With the deepening of research, it is expected to be the main digestive tract reconstruction method after radical resection of proximal gastric cancer.

##### Jejunal Pouch Interposition

Several studies have confirmed that interposed jejunal pouch has a good anti-reflux effect (24, 45) and reduces the tension of the anastomosis between esophagus and remnant stomach, which is safer. In addition to efficacious RE prevention, another big merit of this reconstruction is that interposed jejunal pouch greatly increases the remnant stomach capacity, which can improve postoperative nutritional status and reduce postoperative weight loss (24, 46). A study has shown that JPI has a great advantage in postoperative food intake (47). Three months after the operation, the single food intake of the JPI group reached more than 80% of the preoperative single food intake, while that of the JI group was less than 50%. The percentage of postoperative weight loss in the JPI group was lower than that in the JI group.

Similar to JI, JPI also has some disadvantages such as more intraoperative blood loss and longer time needed resulting from the complicated jejunal pouch making (19). Moreover, food siltation in pouch may be another concern that needs to be addressed after JPI. A study found that JPI is superior to other reconstruction methods in terms of postoperative nutritional status and reducing the number of daily meals (48). But, the postoperative barium meal shows that the jejunal pouch has severe swelling, suggesting that food siltation may occur in the pouch under this procedure, which is similar to the results of another study (49). In these two studies, the complication of JPI was food siltation in the pouch and ineffective conservative treatment. DTR was adopted for the second operation. The reason for this complication is considered to be the irreversible expansion of the jejunal pouch due to frequent excessive food intake in single meal. Based on these results, we can draw the conclusion that while a large single meal intake is an advantage of JPI, it may also increase the risk of food siltation in the pouch. Therefore, it is necessary for surgeons to remind patients (especially young patients) who undergo JPI after PG to avoid excessive food intake in a single meal.

As a digestive tract reconstruction method after PG, the interposed pouch not only can effectively prevent postoperative complications such as RE, but can also increase the capacity of the remnant stomach for single postoperative food intake, and thus improve the postoperative nutritional status. If clinical surgeons can remind patients not to intake excessive food in a single meal after surgery, this method may be an optimal choice for digestive tract reconstruction after PG.

##### Double Tract Reconstruction

DTR can increase the reservoir volume and reduce the occurrence of RE (25, 26, 50, 51). Chyme slowly enters the small intestine, which can reduce the occurrence of dumping syndrome, and food can smoothly enter jejunum from the two pathways of remnant stomach and interposed jejunum, so the incidence of gastric emptying disorders is also significantly reduced. Because of the shunting effect of interposed jejunal pathway, the amount of food entering remnant stomach is reduced, so the stimulation of the gastric antrum by food is also alleviated, and thus the secretion of gastric acid is decreased. Therefore, the proportion of patients who need to take proton pump inhibitors after PG-DTR is also significantly smaller (51, 52).

Several studies on the comparison of complications and nutritional status after laparoscopic EG and laparoscopic DTR showed that the incidence of reflux symptoms and anastomotic stenosis in the laparoscopic EG group was significantly higher than that in the laparoscopic DTR group (p < 0.001) (26, 50, 51). Regarding postoperative nutritional status, the reduction rate of cholinesterase in the laparoscopic EG group was significantly higher than that in laparoscopic DTR group (p = 0.008) (50). This study shows that compared with the traditional EG, DTR has better outcomes in short-term postoperative nutritional status and prevention of gastroesophageal reflux and anastomotic stenosis. These findings indicate DTR may be an effective way to reconstruct the digestive tract after laparoscopic PG.

In 2020, a study comparing DTR and EG after robotic PG was reported (52). In this study, no patients in the DTR group had complications of Clavin–Dindo grade II or above. These outcomes indicated DTR may be able to reduce the occurrence of RE after surgery. The two groups were similar in food intake after discharge, but nutritional indicators such as the rate of postoperative/preoperative weight and albumin level three months after the operation in DTR group were higher than in EG group. This study suggests the DTR method following a fully robotic PG may get more benefits with regard to short-term postoperative results.

As mentioned above, it has been proven that PG with DTR has advantages in reducing the incidence of RE, anastomotic stenosis, dumping syndrome, gastric emptying disorder and improving the nutritional status of patients after surgery, whether under open, laparoscopic, or robotic. It is not difficult to see that DTR has considerable potential applications after PG.

##### Modified Double Tract Reconstruction

Nomura et al. have more research on this procedure. His team conducted two comparative studies of modified DTR and DTR in 2014 and 2018. In the 2014 study, the incidence of reflux esophagitis in both groups was 10%, which was lower than direct anastomosis between esophagus and remnant stomach (27). Endoscopic examinations were able to reach the remnant stomach smoothly. They also found the postoperative/preoperative weight ratio in the modified DTR group was significantly higher than in the DTR group. In the DTR group, the plasma acetaminophen concentration and insulin level increased significantly at 15 and 30 min after oral administration, while the blood glucose level increased at 30 and 60 min, and the increasing trend was more moderate than the modified DTR group. Based on the above results, the study concluded that although modified DTR is an ideal way to preserve gastric function and maintain weight after PG, DTR seems more suitable for proximal gastric cancer patients with impaired glucose tolerance.

The team published another study on the comparison of modified DTR, DTR, and total gastrectomy with Roux-en-Y anastomosis in 2018 (28). The results suggested that the postoperative/preoperative weight ratio of the DTR group and modified DTR group after PG was higher than that of TG. The level of acetaminophen after laparoscopic PG was significantly lower than that after laparoscopic TG. It is worth noting that the levels of acetaminophen, insulin, and gastrin in laparoscopic modified DTR group were significantly higher in the sitting position than in the supine position, while the laparoscopic DTR group and the laparoscopic TG group were more stable in the two positions. Therefore, this study concluded that laparoscopic DTR can maintain smooth intestinal absorption of nutrients and improve QOL. In laparoscopic DTR group, postural changes have little influence on intestinal absorption of nutrients and hormone secretion in comparison to modified DTR, so it may be the reconstruction method that provides the most stable results.

Findings in these two articles provide a new train of thought for the choice of reconstruction method for gastric cancer patients after PG, that is, individualized choice of the most suitable reconstruction method after PG according to situation of the patient. We support the view that surgeons should not completely rely on guidelines. Guidelines derived from evidence-based medicine aim to standardize the treatment process and improve the detection and treatment of the entire medical community. However, we should know that improving the overall medical level is not equal to improving the medical level of every individual. The authority and practicality of all kinds of guidelines are indisputable, and the guidelines have indeed improved the diagnosis and treatment of different diseases. But in practice, it cannot guarantee that all patients will receive correct and timely treatment when doctors follow the guidelines exactly regardless of the individual differences of the patient. For example, although modified DTR is more ideal than DTR in preserving stomach function and maintaining weight after PG, DTR seems more suitable for patients who have impaired glucose tolerance for the reason that the increasing trend of blood glucose after meal is more moderate in the DTR group than in the modified DTR group.

## Oncological Safety

Many studies have proved PG has similar oncological outcomes with TG in recent years (5, 7, 14, 53). In Jung Ko’s study, PG-DTR was even associated with better survival outcomes than the TG-RY group (54). But it is worth noting that the PG-DTR group had smaller tumors than the TG group (P = 0.02). And most of the seven TG patients who died were elderly, which may be owing to the poorer nutritional status of TG than PG. Several meta-analysis research studies have also confirmed that the 5-year overall survival rate of PG was similar or even better when compared to that of TG (55–57).

It is well known that oncological safety is a priority factor when comparing different surgical methods. Currently, the oncological safety of proximal gastrectomy has been recognized by gastrointestinal surgeons. However, at present, there are very few studies comparing oncological safety of different reconstruction methods after PG.

According to the results of a study conducted by Tokunaga comparing the outcomes of patients after PG with JI and PG with EG, there was no significant difference in the 5-year survival rate between the EG group (94.2%) and the JI group (96.9%) (18). In Chen’s study, there was also no significant difference in local tumor recurrence (p = 1.000) between the EG group and the tube-like stomach EG group in the 1 year follow-up visits (20). The results of several studies included in this article comparing the 5-year overall survival rates of TG with PG are listed in **Table 3**. To our knowledge, no study has shown that various alimentary tract reconstruction methods after PG have significant differences in oncological safety. We look forward to large multicenter clinical trials with longer follow-up comparing the overall survival and long-term prognosis of various reconstruction methods after PG.

**Table 3:** The 5-year overall survival rates (%) of Roux-en-Y anastomosis after TG and various kinds of reconstruction methods after PG.

Author	Country	Year	TG	PG				P-value
				Traditional EG	Tube-like stomach EG	JI	DTR	
Ichikawa (5)	Japan	2014	95.0	97.0	\	\	\	0.86
Masuzawa (14)	Janpan	2013	99.1	94.0	\	94.4	\	\
Tokunaga (18)	Japan	2008	\	94.2	\	96.9	\	ns
An (53)	Korea	2007	99.2	\	98.5	\	\	0.57
Ko (54)	Korea	2019	81.6	\	\	\	100.0	0.02
Son (16)	Korea	2014	95.3	95.6				0.79
Yamasaki (7)	Janpan	2020	92.0 (3 year)	96.0 (3 year)				0.49

ns, no significant difference.

## Discussion

With the increasing incidence of proximal gastric cancer, surgeons are paying more attention to proximal gastric cancer. TG with D2 lymph node dissection was considered to be the standard surgery in the past several decades for it can avoid severe RE and radically sweep away the possible metastatic lymph nodes in the distal gastric region. But the poor QOL caused by poor postoperative nutritional condition after TG is a real concern for patients and surgeons. In this context, PG has attracted more attention in recent years.

PG is indicated for upper third stomach tumors and Siewert III adenocarcinoma of the esophagogastric junction. It is worth noting that Siewert III cancers resemble strictly gastric cancers of the upper third, but they are difficult to discover since they are normally diagnosed only in advanced stage. Moreover, surgeons can also perform PG for a benign tumor or GIST. Due to its ability to preserve part of the stomach function and improve the postoperative nutritional status of patients, PG has been increasingly used in proximal gastric cancer patients.

It is well known that every coin has two sides. While preserving the distal stomach to improve the postoperative nutritional status of patients, PG with traditional EG significantly increases the incidence of RE due to the preservation of the distal stomach. Traditional EG is the simplest method among them, but its incidence of RE and anastomotic-related complications such as stenosis and leakage is higher than that of other reconstruction methods (18, 19, 29). Therefore, various kinds of functional digestive tract reconstruction methods have been proposed to solve the postoperative complications of PG, especially RE.

Tube-like stomach EG after PG is not only simple and safe but also has a considerable effect on preventing RE (21). Compared with other reconstruction methods, the anatomical structure of the tube-like stomach has many theoretical advantages. However, a study suggests excessive resection and suture during the process of tube-like stomach making will lead to poor healing of anastomosis and postoperative anastomotic-related complications (36). Therefore, attention should be paid to the preservation of blood vessels such as the right gastric vessels and the right gastroepiploic vessels, which may provide affluent blood to the tissue to reduce anastomotic-related complications.

Many studies have shown huge advantages of DFT such as preventing postoperative RE and having a lower risk of anastomotic leakage (22, 23, 38, 39). Because this method uses muscle flaps to increase the pressure on lower esophagus to prevent RE, patients may experience postoperative anastomotic stenosis due to excessive muscle flap tension during the operation. Therefore, surgeons should attach more importance to controlling the muscle flap tension during anastomosis to find a balance between the prevention of reflux esophagitis and anastomotic stenosis.

It is considered that the reflux prevention mechanism of SOFY is a consequence of the combination of valvuloplasty and fundoplication. Although there is very little research on SOFY, according to the results of Yamashita’s study (12), SOFY is simple to perform and able to overcome postoperative RE and anastomotic-related complications such as stenosis and leakage. So this method may have wide clinical application prospect, and we look forward to large sample multi-center randomized controlled trial to generate more convincing data with regard to SOFY.

The clinical application of JI after PG has increased significantly in recent years. A lot of studies have confirmed the safety of this procedure and it excellent anti-reflux effect (18, 19, 42). At present, the main controversy about jejunum interposition is the length of the interposed jejunum. Because the sample size of existing studies about the optimal length of interposed jejunum are relatively small, a large sample prospective randomized controlled study is needed to guide clinical practice. Although the previous study showed JI has some problems, such as functional disorders and technically complicated nature of the procedure (22, 38), it should be a prior choice for digestive tract reconstruction for it could effectively prevent severe RE, and it is expected to be the main digestive tract reconstruction method after radical resection of proximal gastric cancer.

JPI was designed on the basis of JI, so there are some similarities between them such as efficacious RE prevention and technically complicated procedure (24, 45). Apart from these characteristics, JPI also has its own merit and demerit. The interposed jejunal pouch greatly increases the remnant stomach capacity, which can increase single meal intake after surgery, improve postoperative nutritional status, and reduce postoperative weight loss (24, 46, 47). While a large single meal intake is a merit of JPI, it may also increase the risk of jejunal pouch swelling (48, 49). Therefore, it is necessary for surgeons to remind patients who undergo JPI to avoid excessive food intake in single meal to prevent food siltation in the pouch. In view of the fact that JPI has better effect in preventing RE and improving postoperative nutritional status, it may be an optimal choice for digestive tract reconstruction after PG.

There is already a lot of evidence from existing studies suggesting DTR can reduce the occurrence of dumping syndrome, gastric emptying disorders, and RE (25, 26, 50, 51). These advantages resulted from two main reasons: one reason is that the interposition of jejunum increases the reservoir volume; and the other is that the shunting effect of the interposed jejunal pathway can alleviate the stimulation of the gastric antrum by food which can decrease the secretion of gastric acid.

With the progress of technology, DTR is gradually being used in laparoscopic or robotic PG with good outcomes. At the same time, some modified procedures based on DTR are gradually being proposed. The most notable method among those modified procedure is modified DTR, which blocks the interposed jejunum pathway below the anastomosis of jejunum with remnant stomach. Relevant research studies show that modified DTR is more ideal than DTR in preserving stomach function and in maintaining weight after proximal gastrectomy (27, 28). It may be attributed to passage of all food through the remnant stomach pathway in modified DTR, which digests and absorbs more fully than the interposed jejunum pathway. However, modified DTR also has some disadvantages such as the increasing trend of blood glucose after meal is steeper than in the DTR group and postural changes have more influence on intestinal absorption of nutrients and hormone secretion in comparison to DTR.

Oncological safety should be a priority when comparing different surgical methods. Unfortunately, there are very few studies comparing oncological safety of different reconstruction methods after PG at present. And so far no study has shown that various alimentary tract reconstruction methods after PG have significant differences in oncological safety. We look forward to large multicenter clinical trials with longer follow-up comparing the overall survival and long-term prognosis of various reconstruction methods after PG.

In conclusion, various functional reconstruction methods have their own advantages and disadvantages. Based on existing studies, the best way to reconstruct digestive tract after PG is undetermined yet. Therefore, many large scale high-level clinical research studies are needed to choose an ideal reconstruction method in the future. At the same time, it is necessary for clinical surgeons to establish a concept in mind, that is, the guidelines supported by studies are just references. In clinical practice, surgeons should consider the condition of the patient for individualized selection of the most appropriate reconstruction method rather than completely relying on the recommendations of relevant guidelines.

## Author Contributions

SBL designed this review and write the manuscript. FM, ZZ, and LP helped to design this review and write the manuscript. WY, JC, CL, and FG assisted in writing and editing the manuscript. SJ, SXL, XC, and YH reviewed and revised the manuscript. All authors contributed to the article and approved the submitted version.

## Funding

This study was supported by the National Cancer Climbing Fund (No.NCC201816B048) and Tackle Key Problems in Medicine of Henan Province (201003124, LHGJ20200188).

## Conflict of Interest

The authors declare that the research was conducted in the absence of any commercial or financial relationships that could be construed as a potential conflict of interest.

## Publisher’s Note

All claims expressed in this article are solely those of the authors and do not necessarily represent those of their affiliated organizations, or those of the publisher, the editors and the reviewers. Any product that may be evaluated in this article, or claim that may be made by its manufacturer, is not guaranteed or endorsed by the publisher.
